# Vitamin D—An Effective Antioxidant in an Animal Model of Progressive Multiple Sclerosis

**DOI:** 10.3390/nu15153309

**Published:** 2023-07-26

**Authors:** Michaela Tanja Haindl, Muammer Üçal, Willibald Wonisch, Michaela Lang, Marta Nowakowska, Milena Z. Adzemovic, Michael Khalil, Christian Enzinger, Sonja Hochmeister

**Affiliations:** 1 Department of Neurology, Medical University of Graz, 8036 Graz, Austria; 2 Department of Neurosurgery, Medical University of Graz, 8036 Graz, Austria; 3 Otto Loewi Research Center, Department of Physiological Medicine, Medical University of Graz, 8010 Graz, Austria; 4 Faculty of Health, University of Applied Sciences Wiener Neustadt, Campus 1, 2700 Wiener Neustadt, Austria; 5 Department of Clinical Neuroscience, Karolinska Institutet, 171 64 Stockholm, Sweden

**Keywords:** vitamin D, progressive multiple sclerosis, animal model, neuroprotection

## Abstract

Vitamin D (VD) is the most discussed antioxidant supplement for multiple sclerosis (MS) patients and many studies suggest correlations between a low VD serum level and onset and progression of the disease. While many studies in animals as well as clinical studies focused on the role of VD in the relapsing-remitting MS, knowledge is rather sparse for the progressive phase of the disease and the development of cortical pathology. In this study, we used our established rat model of cortical inflammatory demyelination, resembling features seen in late progressive MS, to address the question about whether VD could have positive effects on reducing cortical pathology, oxidative stress, and neurofilament light chain (NfL) serum levels. For this purpose, we used male Dark Agouti (DA) rats, with one group being supplemented with VD (400 IE per week; VD^+^) from the weaning on at age three weeks; the other group received standard rodent food. The rat brains were assessed using immunohistochemical markers against demyelination, microglial activation, apoptosis, neurons, neurofilament, and reactive astrocytes. To evaluate the effect of VD on oxidative stress and the antioxidant capacity, we used two different oxidized lipid markers (anti- Cu^++^ and HOCl oxidized LDL antibodies) along with colorimetric methods for protective polyphenols (PP) and total antioxidative capacity (TAC). NfL serum levels of VD^+^ and VD^−^ animals were analyzed by fourth generation single-molecule array (SIMOA) analysis. We found significant differences between the VD^+^ and VD^−^ animals both in histopathology as well as in all serum markers. Myelin loss and microglial activation is lower in VD^+^ animals and the number of apoptotic cells is significantly reduced with a higher neuronal survival. VD^+^ animals show significantly lower NfL serum levels, a higher TAC, and more PP. Additionally, there is a significant reduction of oxidized lipid markers in animals under VD supplementation. Our data thus show a positive effect of VD on cellular features of cortical pathology in our animal model, presumably due to protection against reactive oxygen species. In this study, VD enhanced remyelination and prevented neuroaxonal and oxidative damage, such as demyelination and neurodegeneration. However, more studies on VD dose relations are required to establish an optimal response while avoiding overdosing.

## 1. Introduction

Multiple sclerosis (MS) is a chronic disease of the central nervous system (CNS) that mainly affects young adults. It is caused by an autoimmune response to central nervous system structures, including both white and grey matter. After decades of research, many pathomechanisms behind this disease are still not fully understood. Despite the availability of immunomodulatory therapeutics, which effectively reduce inflammatory processes directed against the CNS, the symptoms still might worsen, which heavily impairs the quality of life of patients. This underlines the importance of identifying novel treatment targets, especially for the progressive disease phase (PMS), which is associated with the development of severe and irreversible disability. Vitamin D (1,25-dihydroxyvitamin D; VD) is the most discussed antioxidant supplement for several autoimmune diseases, including MS. Its functions comprise regulation of calcium homeostasis as well as effects on immune response. VD is not only assigned to the environmental risk factors for developing MS, but is also part of a genetic factor and an epigenetic factor [1,2]. The association between VD receptor single-nucleotide polymorphisms and MS risk has been reported by many authors, with a few studies producing opposite results [3]. Oxidative stress is discussed as a mediator of demyelination and axonal damage in both MS and respective animal models [4] and VD seems to act as a regulating factor on oxidative stress [5]. Many studies, especially in relapsing-remitting MS, suggest a positive effect of higher VD serum levels on onset, disease activity, and progression of the disease, leading to the idea of using VD as a supplementary therapy for MS patients. Several clinical trials and research in experimental animal models of MS addressed this question, resulting in mixed and sometimes even contradictory data [6,7,8]. The design of clinical trials can be especially problematic due to the possibility of overdose. Thus, some studies focused on safe VD supplementation and, in clinical trials, add-on therapy for short-term periods with doses up to 40,000 IU per day were considered safe. Due to the still existing knowledge gap for long-term and high-dose therapy, the supplementation of VD in MS is still a sensitive task that needs to be supervised by physicians [9,10]. Nevertheless, when applied correctly, VD could have high potential in MS therapy. Especially in PMS, establishing novel treatment approaches is an unmet need, as most available MS therapeutics are only effective in the relapsing-remitting disease phase. Similarly, much more research results for the safe use of VD supplementation are available for the relapsing-remitting MS compared to the progressive disease phase. Thus, the potential and possible effect of VD on this disease form or on preventing progression remains widely unclear. In the literature, there are only a few associations between low VD status and early conversion to the secondary progressive MS phase, pointing towards the role of VD in regulating immune responses against the CNS [11]. Other studies only compared a few outcomes (associations between VD serum level and imaging data) in PMS patients—without comparing to a corresponding VD-supplemented patient group—with the conclusion that there is no association between VD levels and visual function or brain volume in PMS [12,13]. Despite the great importance of further clinical trials to evaluate the potential of VD as an MS treatment, one big issue is the heterogeneity of the disease, genetic factors, and, of course, differences in sunlight exposure. Thus, experimental animal models are a valuable and important tool for the basic research of cellular mechanisms to achieve standardized conditions.

Since cortical pathology is sparse or absent in common MS animal models, such as experimental autoimmune encephalomyelitis, our research team developed a rat model that reassembled cellular features of the PMS very well [14]. In the present study, we used this model to gain more insight into the effect of VD on cortical pathology. We focus on VD as an antioxidant through analysis of total antioxidant capacity (TAC), a parameter that is modulated either by radical overload or by supplementation of antioxidants [15]. Additionally, we measured the polyphenol (PP) concentration in the serum of our animals, which are compounds that are potentially valuable for modulating local and systemic inflammatory environments [16,17]. Histologically, we comprehensively investigated two different oxidized lipids (via anti- Cu^++^ and HOCl oxidized LDL antibodies).

Furthermore, we assessed the effect of VD on neurofilament light chain (NfL) serum levels as a marker of axonal damage in the rats’ sera through high-sensitivity single-molecule array (SIMOA) quantitation [18].

All analyses were correlated to thorough histopathological evaluation to assess differences between VD-supplemented (VD^+^) and not supplemented (VD^−^) animals (controls).

## 2. Materials & Methods

### 2.1. Animals

In total, 45 male Dark Agouti (DA) rats aged 10–12 weeks, obtained from Janvier, France, underwent the protocol described in detail in Ücal et al. 2017 [14]. Animals were divided into VD-supplemented (*n* = 22) and not supplemented (*n* = 23) groups. Table 1 shows a detailed list of the animals used and their groups. An overview with corresponding schemata is given in supplementary Figure S1. Rats with implanted catheter only and no further treatments were used as healthy appearing control animals (HAC). All animal experiments were carried out under approval of the local authorities (Federal Ministry of Science and Research; 66.010/0072-WF/V/3b/2017).

### 2.2. Experimental Setup

All animals underwent the experimental protocol described in detail in Ücal et al. 2017 to elicit cortical pathology. The VD animal group orally received one drop of VD solution (=400 IE Vitamin D; Fresenius-Kabi, Graz, Austria) per week, starting from the weaning (at age of 3 weeks) until the end of the experiment (=VD^+^ group; *n* = 22). All other groups received standard rodent food only (=VD^−^ group; *n* = 23). Briefly, the animal model starts with the catheter implantation. After a healing period of two weeks, all rats are immunized with myelin oligodendrocyte glycoprotein (MOG) in incomplete Freund’s adjuvants. The MOG antibody titer is validated via ELISA after four weeks. Once the titer is sufficient, animals receive 2 µL of a cytokine mixture of interferon gamma (IFN-γ) and tumor necrosis factor alpha (TNF-α) via a programmable syringe pump through the catheter in order to open the blood–brain barrier. The peak of cortical pathology can be observed on day (d) 15, and on d30 the first traces of remyelination are detectable, and even a “second relapse” can be generated by an additional cytokine injection on d30—indicated as d45* [14].

### 2.3. Blood Sampling, Euthanasia, and Tissue Extraction

Blood sampling was performed before catheter implantation (HAC), after MOG immunization, and on d1, d3, d15, d30, and d45* after cytokine injection. NfL was additionally measured in the sera of animals one day after catheter implantation. Serum was harvested by centrifugation at 4600 rpm two times, one hour after blood sampling, and was stored at −70 °C until use according to guidelines [19]. Animals were sacrificed on d15, d30, and d45* according to the protocol described in detail in Ücal et al. 2017 [14]. Briefly, anesthesia was induced with 4% isoflurane followed by cardial injection of 25 mg Thiopental (Sandoz, Kundl, Austria). After reaching deep anesthesia, animals were transcardially perfused with 4% paraformaldehyde (PFA; Merck, Darmstadt, Germany) in phosphate-buffered saline (PBS, pH = 7.4). Brains and spinal cords were dissected and post-fixed in 4% PFA for 24 h. Only brains were used for further detailed histopathological analysis, as spinal cords are unaffected in this animal model and are only routinely checked [14].

### 2.4. Neuropathology and Immunohistochemistry

After embedding in paraffin, brain tissue was cut into 1.5 µm sections. For immunohistochemical (IHC) staining, sections were dewaxed in xylene (Fisher Thermo Scientific, Schwerte, Germany), rehydrated, and steamed for 1 h in citric acid (Merck) [1,14]. After incubation with 2.5% normal horse serum (Vector Laboratories Burlingame, Newark, CA, USA) for 20 min at room temperature, sections were covered with primary antibodies and incubated overnight at 4 °C. A detailed list of the used antibodies and respective dilutions is given in supplementary Table S1. The ImmPRESS System (Vector Lab., secondary antibodies) was used, visualized with 3,3′diaminobenzidine-tetrahydrochloride (DAB, Sigma-Aldrich, Buchs, Switzerland), and counterstained with hematoxylin. After dehydration, slides were covered with Shandon Consul-Mount (Fisher Thermo Scientific) and a coverslip.

### 2.5. Quantitative Histopathological Evaluation

One investigator blinded for experimental groups assessed the quantification of demyelination, microglial activation, apoptotic cells, neuronal cell loss, and histological oxidative stress markers with an optical grid at a magnification of 20× on the microscope (Zeiss AXIO Imager.M2). Demyelination was assessed by counting the loss of proteolipid protein (PLP) immunoreactivity, and values were then transformed to PLP loss/mm^2^. Activated microglia (Iba1), apoptotic cells (Caspase-3), neurons (NeuN), and oxidative stress markers (Cu^++^ oxidized LDL and HOCl oxidized LDL) were assessed in three full optical grids in the cortex per hemisphere with the 20× objective. Average values were converted to cells/mm^2^.

### 2.6. Colorimetric Methods for Antioxidative Capacity and Polyphenols

To evaluate antioxidative effects, we used two different test systems, i.e., the total antioxidative capacity (TAC^®^; Omnignostica Ltd., Höflein an der Donau, Austria) and Polyphenols (PPm^®^; Omnignostica Ltd., Austria) as described previously [20,21]. All serum samples were analyzed in double. The TAC assay was performed according to the manufacturer’s instructions with distinct modifications for measuring small sample volumes. In brief, 10 µL standards, controls, and samples (undiluted) were pipetted into a 96-well microtiter plate. After that, 40 µL of reagent A was added into all wells within 1 min. Subsequently, 20 µL of reagent B was added to all wells within another minute. After an incubation time of precisely 20 min at 4 °C, the experiment was stopped by adding 20 µL of stop solution and the absorbance was measured at 450 nm using a microplate reader. The PP test was performed per the manufacturer’s instructions; no modifications were required.

### 2.7. NfL Measurement Via Single-Molecule Array (SIMOA)

NfL was measured with a commercial ultrasensitive SIMOA NF-light assay on a SR-X analyzer (Quanterix, Billerica, MA, USA), based on single-molecule arrays and simultaneous counting of single captured microscopic beads carrying antibody complexes. The analytical sensitivity of this technique is manifold higher than that obtained with conventional photometric test systems, thus enabling a reliable measurement of low NfL concentrations in blood samples [18]. Advanced SIMOA NfL-kits (Quanterix, MA, USA) are used for clinical questions. Fortunately, there is a high cross-reactivity for rat serum, and we could use those advanced kits in this study according to the manufacturer’s instructions.

### 2.8. Statistical Analysis

Statistical analysis was performed using SPSS Statistics (v23, IBM, Armonk, NY, USA), and graphs were illustrated in Microsoft Excel 2010 as box-plots. The data was checked for normal distribution via the Kolmogorov–Smirnov test; we used non-parametric tests in all cases. The text body provides median values and interquartile range (IQR). We used the Kruskal–Wallis test followed by Mann–Whitney U test for statistical significance testing. A difference of *p* < 0.05 was considered to be statistically significant. All statistical tests performed for each of the figures are summarized in supplementary Table S2.

## 3. Results

### 3.1. VD^+^ Animals Show a Better Preservation of Cortical Cellular Structures and Less Microglia Activation

**Figure 1:**
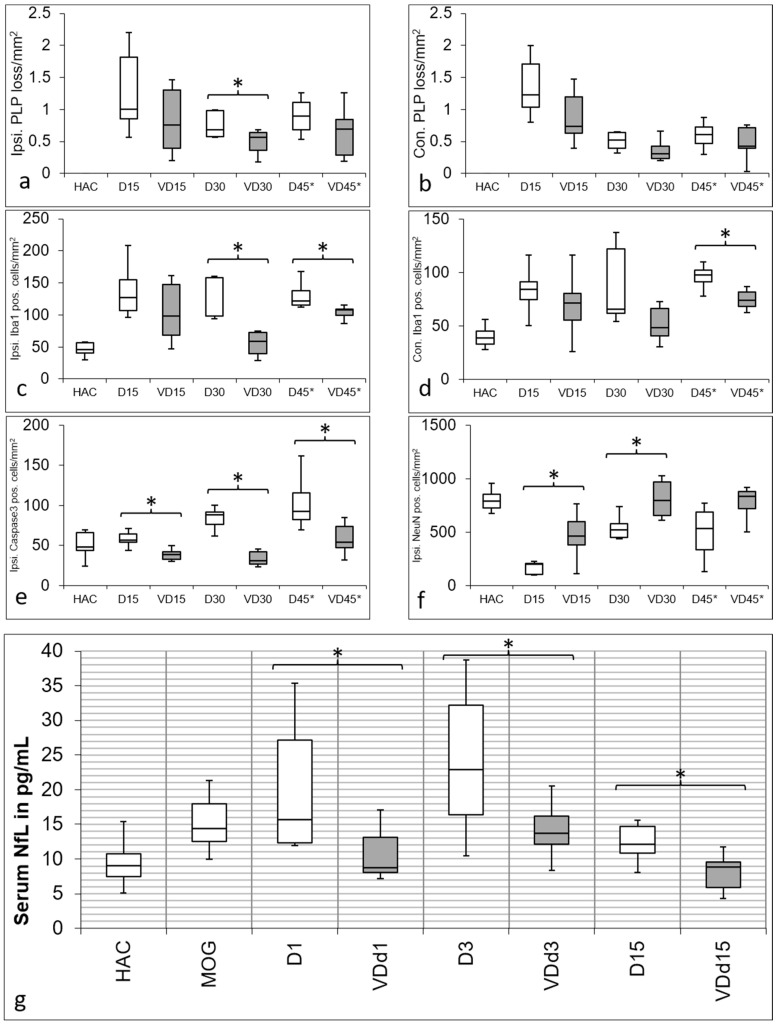
Quantification of IHC staining. PLP loss is displayed in the first row in (**a**) on the ipsilateral and (**b**) contralateral side with a similar pattern. No cortical demyelination is detectable in HAC, and demyelination is most pronounced in d15 animals, with more PLP preservation in VD^+^ animals. Also, d30 (*p* = 0.05) and d45* animals show more PLP loss when not supplemented with VD. Iba1 quantification is shown in (**c**) on the ipsilateral side and (**d**) contralateral side with a comparable trend. Microglial activation is lower overall in VD^+^ animals with a significant difference on the ipsilateral side on d30 (*p* = 0.004) and d45* (*p* = 0.018), as well as on the contralateral side on d45* (*p* = 0.038). Quantification of apoptotic cells is shown in (**e**) on the ipsilateral side, with a significant reduction on d15 (*p* = 0.002), d30 (*p* = 0.004) and d45* (*p* = 0.023) in VD^+^ animals. The preservation of neurons in VD^+^ animals is shown in (**f**) on the ipsilateral side, with a significant result on d15 (*p* = 0.019) and d30 (*p* = 0.019). Asterisks indicate significant differences. In diagram (**g**), there is a significant increase of NfL detectable after MOG immunization compared with HAC (*p* < 0.001). In comparison to HAC, all VD− animals showed a significant increase in NfL serum levels (d1, *p* < 0.001; d3 *p* < 0.001; d15 *p* = 0.020). This pattern changes in VD+ animals, where only d3 animals differ significantly from HAC (*p* = 0.008). Although all groups show a similar trend over d3 to d15 after cytokine injection, VD+ animals have significantly lower NfL serum levels on all days (d1, *p* = 0.027; d3, *p* = 0.033; d15, *p* = 0.006). Asterisks indicate significant differences.

The quantification of PLP loss is displayed in Figure 1 (a; ipsilateral side, and b; contralateral side). Overall, the PLP loss is lower in VD^+^ animals, although reaching statistical significance only on d30 on the ipsilateral side (*p* = 0.05). There is also a significant difference in microglial activation detectable on the ipsilateral side on d30 (*p* = 0.004) and d45* (*p* = 0.018) (Figure 1c), and on the contralateral side (Figure 1d) on d45* (*p* = 0.038). Furthermore, there is no significant difference between HAC and VD d30 animals on both the ipsilateral and contralateral sides. Apoptotic cells are significantly reduced on the ipsilateral side on d15 (*p* = 0.002), d30 (*p* = 0.004), and d45* (*p* = 0.023) (Figure 1e). There is also a significantly better neuronal preservation on d15 (*p* = 0.019) and d30 between VD^+^ and VD^−^ animals (*p* = 0.019), which is displayed in Figure 1f.

**Figure 2:**
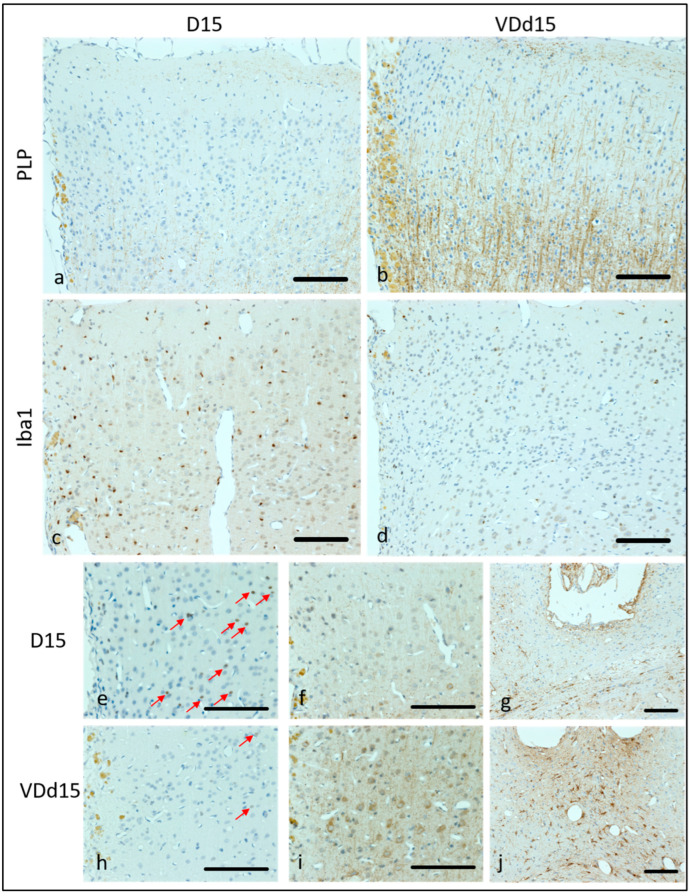
Representative microscopic pictures of IHC staining. All pictures represent the upper right corner of the catheter puncture, except (**g**,**j**), where the area underneath the puncture is displayed. In (**a**,**b**), the PLP staining is shown with much more PLP (brown fibers) remaining in VD^+^ animals. In (**c**,**d**), Iba1 positive cells (small brown dots) represent the microglial activation, which is higher in VD^−^ animals (**c**). D15, VD^−^ animals are shown in (**e**–**g**) compared to VD^+^ d15 animals (**h**–**j**). Caspase-3 staining (apoptotic cells) appears more frequently in VD^−^ animals (**e**) than in VD^+^ ones (**h**). Red arrows indicate apoptotic cells. Neurofilament structures are more preserved in VD^+^ animals (**i**) than in VD^−^ animals (**f**). More reactive astrocytes are detectable underneath the catheter puncture in VD^+^ animals (**j**) compared to VD^−^ ones (**g**). Scalebars represent 100 µm.

Representative microscopic pictures of histological results are displayed in Figure 2, and all scale bars represent 100 µm. In Figure 2a, the PLP loss of a representative d15 animal is shown with hardly any PLP fibers remaining. In comparison, Figure 2b shows a VD-supplemented d15 animal, where the PLP structures are still clearly visible. The microglial activation is much higher in d15 animals (Figure 2c) than in VD^+^ animals (Figure 2d). Apoptotic cells are also reduced in VD^+^ animals on d15 (Figure 2e, VD^−^, h, VD^+^). Neurofilament structures are better preserved in VD-supplemented animals in comparison to normal d15 animals (Figure 2f,i). Astrocytic reactions are more pronounced in VD^+^ animals (Figure 2g,j).

### 3.2. NfL Serum Level Is Lower in VD^+^ Animals, Representing Less Axonal Loss

As expected, the catheter implantation itself pushes the NfL serum levels to an immense increase of 356 IQR 216 pg/mL (not shown in the diagram) compared to the HAC with 9.0 IQR3.3 pg/mL. This value, caused by the mechanical trauma, alleviates during the healing period and reaches a value of 14.4 IQR 5.5 pg/mL after MOG immunization. There is a significantly lower NfL concentration detectable in VD^+^ animals on d1 (*p* = 0.027) d3 (0.033) and d15 (0.006) after cytokine injection (Figure 1g). VD^+^ animals thereby follow the same pattern as it is shown for the VD^−^ animals, with the highest NfL serum levels on d3, but in a significantly lower range.

### 3.3. Histological Oxidative Stress Markers Are Lower in VD^+^ Animals

**Figure 3:**
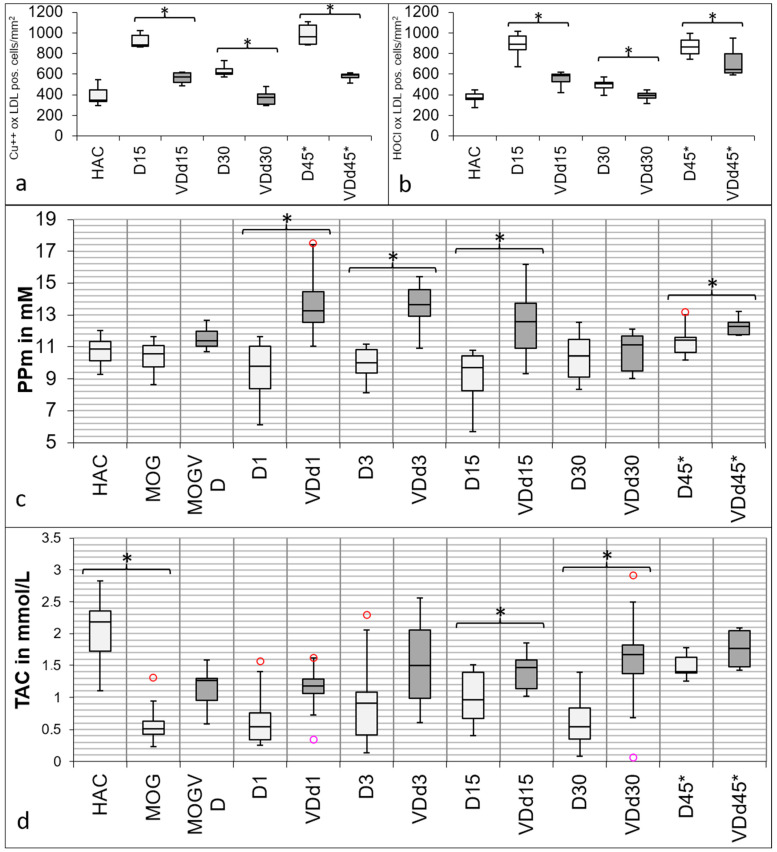
Differences in oxidative stress markers in histology and serum between VD^+^ and VD^−^ rats. Both oxidative stress markers show significant differences between VD^+^ and VD^−^ rats. In (**a**), the quantification of IHC-positive stained Cu^++^-oxLDL cells is shown, and in (**b**), the quantification of IHC-positive stained HOCl-oxLDL cells is displayed. There is a conspicuous reduction of both markers in VD^+^ animals detectable on d15 (Cu^++^-oxLDL *p* = 0.002; HOCl-oxLDL *p* < 0.001), d30 (Cu^++^-oxLDL *p* = 0.004; HOCl-oxLDL *p* = 0.042), and on d45* (Cu^++^-oxLDL *p* < 0.001; HOCl-oxLDL *p* < 0.001). In (**c**), the differences in protective PP in the serum between VD^+^ and VD^−^ rats are shown. There are no significant differences between HAC and MOG-immunized animals. There is always a significant difference detectable between VD^+^ and VD^-^, except for d30 (d1, d3, and d15 *p* < 0.001; d45* *p* = 0.025). In (**d**), the TAC results are shown. There is a significant difference between HAC sera and sera after MOG immunization detectable (*p* < 0.001). Overall, VD^+^ animals have a higher TAC. There is no significant difference detectable between the groups on d3 and d45*, but the groups differ significantly on d15 (*p* < 0.05) and d30 (*p* < 0.040) from each other. Circles indicate minimum and maximum outliers. Asterisks indicate significant differences.

Both markers for oxidative stress show a similar trend towards the experimental groups (Figure 3a,b) with a peak in d15 animals and d45* animals. Overall, there is a significant reduction in VD^+^ animals detectable in both markers on d15 (Cu^++^-oxLDL *p* = 0.002; HOCl-oxLDL *p* < 0.001), d30 (Cu^++^-oxLDL *p* = 0.004; HOCl-oxLDL *p* = 0.042), and on d45* (Cu^++^-oxLDL *p* < 0.001; HOCl-oxLDL *p* < 0.001).

### 3.4. Total Antioxidant Capacity and Polyphenols Are Increased in the Sera of VD^+^ Animals

Figure 3c shows the results of protective PP in serum. The immunization with MOG causes no change in the animals’ baseline serum (HAC) PP level. On d1, there is a significant difference detectable between VD^+^ and VD^−^ (*p* < 0.001), also remaining on d3 (*p* < 0.001) and d15 (*p* < 0.001) after cytokine injection. On d30, there is no significant difference detectable anymore. This changes again on d45*, where the difference between VD^+^ and VD^−^ becomes significant again (*p* < 0.025). In Figure 3d, the results of TAC are displayed. Other than PP, a significant difference is detectable between HAC and MOG-immunized serum (*p* < 0.001). On d1 and d3, there is no significant difference detectable in TAC; unlike d15, where the groups differ significantly from each other (*p* < 0.05). On d30, there is also a significant difference detectable (*p* < 0.040), which is no longer detectable on d45*.

## 4. Discussion

Overall, our data indicate a positive effect of VD supplementation in preserving cortical structures and regulating oxidative stress in our animal model of PMS. At least in our experiment, the most significant effect of VD supplementation in preventing cortical pathology is less pronounced microglial activation, fewer apoptotic cells, and increased preservation of neurons. Moreover, a tendency towards better preservation of PLP and neurofilament structures was observed in association with VD supplementation. On d30, at the start of remyelination in our animal model [14], all investigated histological markers show a significant difference between the VD^+^ and the VD^−^ group. Our finding of significantly more PLP preservation in VD^+^ animals on d30, where remyelination starts in our model, correlates with the conclusion of other studies that VD has a positive effect on remyelination [22].

Another marker investigated during this study is serum NfL, the most promising neurofilament subunit to track neuroaxonal damage [18]. Our data show a high increase of NfL serum levels after one day of catheter implantation before alignment to baseline levels again after the healing period, which is consistent with the acute surgical trauma [23]. After cytokine injection, right at the opening of the blood–brain barrier and at the start of acute cortical demyelination, there is a significant increase of NfL on d1 and d3 again. On d15, when maximum cortical demyelination has been accomplished, NfL decreases again to levels comparable to HAC. Histological data show a peak of cortical pathology with pronounced demyelination on d15 [14], while NfL peaks much earlier, already on d3. This leads to the conclusion that the NfL increase reflects the actively ongoing tissue damage, not the extent of completed cortical demyelination. In our animal cohort, a similar pattern was detectable in the VD^+^ animals but at a significantly lower level on all investigated days. Since NfL rises upon neuroaxonal damage, we conclude that VD supplementation preserved neuroaxonal cell structures in our animal model, although not fully suppressing the pathology.

Precise mechanisms that drive the disease in patients with progressive MS are currently unknown, but demyelination may be triggered by mitochondrial injury from oxidative stress. In MS, it seems to be mainly driven by oxidative bursts in microglia [4,24]. Reactive oxygen species (ROS), if produced in excess—leading to oxidative stress—are suggested to be mediators of demyelination and axonal damage in both MS and associated animal models [4]. The possible links between MS and an imbalance of oxidant/antioxidant cell function may be supported by increased lipid peroxidation products in blood and CSF, and an abnormal expression of heat shock proteins in oligodendrocytes. Under normal circumstances, the potentially damaging effects of ROS are limited by the endogenous antioxidant defenses in the body. This theory is supported by decreased glutathione and tocopherol concentrations and increased uric acid levels observed in demyelinating plaques of MS patients [15]. Consequently, oxidative stress also plays a role in our animal model. Most studies addressing the relevance of oxidative stress for MS progression have focused on brain intrinsic cells generating ROS. Further results from autopsy studies showed that in lesions of white matter and cerebral cortex, demyelination and neurodegeneration are associated with the presence of oxidized lipids [4]. We investigated two different oxidized lipids (Cu^++^-oxLDL and HOCl-oxLDL), and both were found in the tissue of our animals, with a much higher occurrence in VD^−^ animals. Especially on d30, where the first remyelination events appear, there is a highly significant difference detectable between the VD^+^ and VD^−^ groups. This again leads to the conclusion that there is a connection between oxidative stress and remyelination, and VD supplementation facilitates the latter.

VD supplementation seems to also have positive effects on the preservation of neuroprotective PP. Since PP have immunomodulatory properties, they are more concentrated in animal serum on days right after the cytokine injection in both groups. In VD^+^ animals, polyphenols are significantly lower on d30 compared to VD^+^ animals on d1, d3, and d15, but increases again on d45* in the serum of animals that received a second cytokine injection on d30. The second cytokine injection on d30 could be a possible reason for the lowered polyphenols on this day.

In addition, the TAC is increased by VD supplementation. Compared to PP data, the MOG immunization is detectable via determining TAC with a decreasing effect. In VD^−^- animals, the TAC stays approximately the same on d1 and starts to rise again during the experiment, with one decrease on d30. This drop can be prevented by supplementation with VD. Furthermore, at peak disease (d15), the TAC is significantly higher in VD-supplemented animals, suggesting an overall preservation of TAC by VD. ROS-mediated effects depend on fine-tuning a multicellular and multi-cascade network that is not yet fully understood. ROS-mediated pathways and cellular effects are involved in immune cell priming in the peripheral lymphoid organs. Since the oxidative brain environment is altered in MS patients [4], research on specific pathways and cell interactions in suitable models could help understand the mechanisms that regulate the formation and progression of lesions.

Furthermore, the measurements of antioxidative capacity and oxidative stress markers, and the direct ROS measurement post-treatment could be interesting additional information. Therefore, we used a biomarker induced by ROS in our study, i.e., we measured peroxides in the serum of our rodents. Although the method sensitively measures in the micromolar range, we did not obtain measurable concentrations, mainly since rodents synthesize their vitamin C autogenously. Furthermore, radicals are very short-lived and not easy to track during long-term experimental setup. Also, the handling during animal experiments could artificially increase ROS. Since we could not detect measurable peroxide levels in serum, we noticed oxidative stress ex vivo using antibodies against oxidatively modified molecules and measured the antioxidative capacity.

Surprisingly, VD^+^ animals appeared to have more pronounced astrocytic reactions than VD^−^ animals. Since astrocytes have manifold functions and can either be protective or detrimental, we hypothesize that there are different phenotypes of astrocytes involved [25]. A detrimental astrocytic reaction would not fit to all the other results obtained in this study.

Although we of course cannot propose a definitive mechanism behind our findings, we can form hypotheses. Ample literature is available showing that VD protects the CNS from inflammation through the modulation at different levels, including cytokines, growth factors, cell signaling, response to oxidative stress, BBB integrity, and cellular trafficking (reviewed in detail in Galoppin et al., 2022) [26]. Modified immune responses induced by VD in the periphery may also protect the CNS from the inflammatory insult by local protection of the blood–brain barrier (BBB). BBB endothelial cells express VDR and VD have been suggested to provide beneficial effects in MS by protecting the BBB’s integrity [27,28]. Even though in our model the BBB is opened artificially via injection of pro-inflammatory cytokines, VD supplementation may result in a faster and more thorough restoration of the BBB’s function, thus alleviating cortical damage. This hypothesis correlates quite well with the findings shown in the work of Galoppin et al. [26].

During development, astrocytes play a major role in the maturation of the BBB and contribute to establishing the immune-privileged state of the CNS by forming a second barrier, called the glia limitans. This structure encapsulates the entire CNS parenchyma and—along with the BBB—prevents the unrestricted entrance of immune cells. As early responders, astrocytes can react promptly to inflammatory stimuli, such as cytokines. Since many studies have shown that astrocytes express VDR, they are most likely able to respond to VD in an autocrine or paracrine manner, and VD supplementation potentially reduces the pro-inflammatory response of astrocytes in the CNS, leading to the better preservation of the cellular architecture in our study [29,30].

Another possible mechanism may involve microglia as abundant immune cells present in active MS lesions playing a critical role in antigen presentation, recruitment of T cells, ROS production, and release of pro-inflammatory cytokines. Through all these actions they may further amplify neuroinflammation. It has been shown that microglia can synthetize calcitriol in vitro and that they express VDR on their surface [24,31]. In our study, microglia activation was significantly reduced in our VD-supplemented animals. Due to the evidence of VD working as a potent antioxidant, cellular preservation might also be supported by overall ROS reduction.

In summary, the possibilities of VD actions are manifold, acting on multiple sites of the inflammatory pathway, and most likely it is a combination of all these modes of action that lead to the overall beneficial effect we find in our animals. Our effect, however, may even be enhanced by the fact that the animals were supplemented with VD starting from when they were three-weeks old and were growing up with sufficient VD levels. It appears likely that the net effect may be less pronounced when VD supplementation is initiated at a later point in life.

Even though a complete translation of the results obtained in animal studies to the pathophysiologic processes seen in humans is not possible, animal models can help overcome the limitations of clinical studies, thereby elucidating cellular mechanisms. Especially for VD studies, this is one crucial aspect due to the difficulties in clinical study design both for the relapsing-remitting MS and for the progressive disease phase, with much more literature being available for the former. Currently, only a few studies are available regarding VD and PMS with a long disease duration, with no detected connections between VD serum levels and clinical outcomes [12,13]. One major limitation of such mechanistic studies, however, is the onset of VD supplementation, which must start very early [1]—also considering the comparably short disease duration. This is, of course, a limitation for the translation of results for the human PMS situation.

To conclude, VD seems to have potential as a supplement in progressive MS, which is why much more research on VD in progressive MS and associated animal models is required.

## Figures and Tables

**Table 1:**
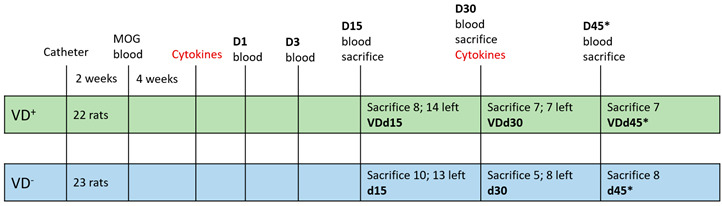
Animal groups and experimental setup. In total, 45 DA rats were used during this experiment, divided into the VD^+^ (*n* = 22) and VD^−^ (*n* = 23) groups (indicated by the two color boxes). Blood was taken (indicated by “blood” on the timeline) before the catheter implantation (HAC), after MOG immunization (“MOG”) and on days 1 to 45 after cytokine injection. The shortcut “d” stands for the day; the number of animals sacrificed in the different groups on the three days is written in the respective box, and the associated group notation is below in bold. The asterisk in group d45* indicates the second cytokine injection on d30.

.	Tissue	Serum		
	Used for IHC	Used for TAC	Used for PP	Used for NfL SIMOA
**HAC**	4	8	16	16
**MOG**	No sacrifice	11	20	10
**VDMOG**	No sacrifice	7	20	n.m.
**d1**	No sacrifice	8	19	8
**VDd1**	No sacrifice	5	15	8
**d3**	No sacrifice	7	12	11
**VDd3**	No sacrifice	11	16	7
**d15**	7	10	14	9
**VDd15**	8	9	20	9
**d30**	5	10	14	n.m.
**VDd30**	7	6	12	n.m.
d45*	7	5	7	n.m.
VDd45*	7	4	7	n.m.
**TOTAL**	**45**	**101**	**192**	**78**

## Data Availability

All original material and data are saved at the Department of Neurology, Medical University of Graz. Please contact the corresponding author for any inquiry.

## Supplementary Materials

The following supporting information can be downloaded at: https://www.mdpi.com/article/10.3390/nu15153309/s1, Figure S1: Schematic overview of the experimental setup and used samples. Table S1: Primary and secondary antibodies used during this study with detailed information; Table S2: Summary of all the statistical tests performed for each of the figures.
