# Supplementary material for: Vitamin D—An Effective Antioxidant in an Animal Model of Progressive Multiple Sclerosis
Source: Nutrients. 2023 Jul 26;15(15):3309. doi: 10.3390/nu15153309 (PMC10421326; doi:10.3390/nu15153309)
Supplement: Supplementary file 1 [file nutrients-15-03309-s001.zip › nutrients-2500079-supplementary.pdf]

Supplementary Material

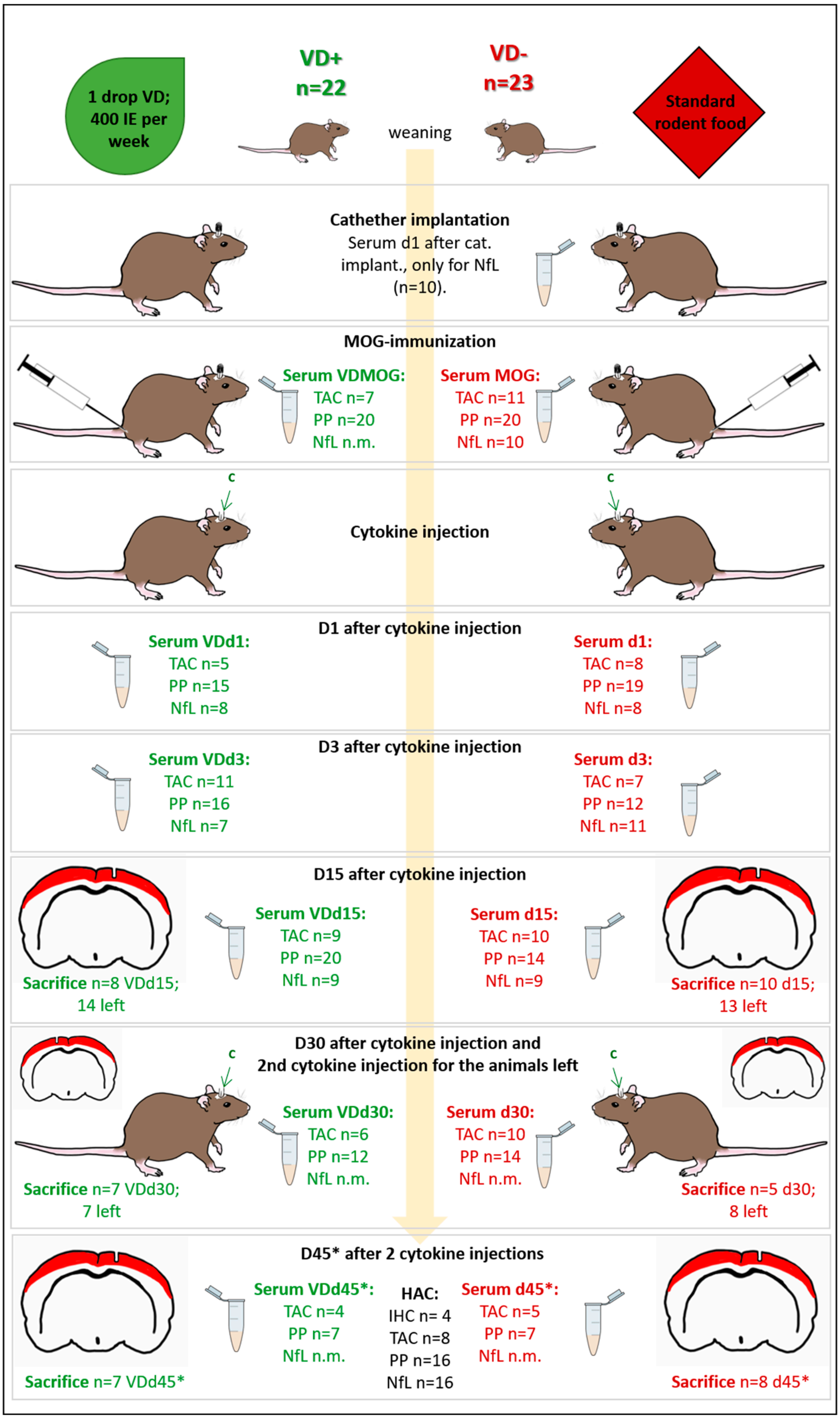

Supplementary Figure S1. Schematic overview of the experimental setup and used samples. In total, 45 DA rats were used during this experiment. The left side represents the VD$^+$ (n=22) group, and the right side represents the VD$^-$ (n=23) group. Following the yellow middle line downwards reveals consecutive steps related to the animal experiment, starting with the weaning and ending with the sacrifice of d45* animals (the asterisk means, that those animals received 2 cytokine injections; one on d0 and one on d30). To the left and right of this line, the harvested material (tissue and serum) is represented with the corresponding animal numbers for each method.

Supplementary Table S1 Primary and secondary antibodies used during this study with detailed information.

Antibody	Target	Host	Dilution	Company	Catalog number	AB ID
Caspase3	Anti-active Caspase3 antibody	rabbit	1:500	Abcam	ab2302	AB_302962
Cu^{++}oxLDL	Anti-lipoprotein (a), oxidized via Cu^{++}	sheep	1:3000		FT-OL11	
GFAP	anti-GFAP Ab-6 (Clone ASTRO6)	mouse	1:100	Thermo Scientific	MS-1376	AB_1095984
HOCloxLDL	Anti-modified LDL (via HOCl)	rabbit	1:2000		HL02	
Iba1	Anti Iba1	rabbit	1:1000	Wako	019-19741	AB_839504
NeuN	Anti-NeuN, clone A60 (KC)	mouse	1:100	Millipore	MAB377-KC	AB_2298772
Neurofilament	Anti-200kD Neurofilament Heavy Chain antibody	rabbit	1:2000	Abcam	ab8135	AB_306298
PLP	Anti-myelin proteolipid protein	mouse	1:500	AbD Serotec	MCA839G	AB_2237198
anti-rabbit ImmPRESS reagent	anti-rabbit IgG	horse	ready to use	Vector	MP-7401	AB_2336529
anti-mouse ImmPRESS reagent	anti-mouse IgG	horse	ready to use	Vector	MP-7422	AB_2336527

Supplementary Table S2 Summary of all the statistical tests performed for each of the figures.

		Check for normal distribution	Significance testing	Significance testing pairwise comparison	Significance level
Figure 1 a	PLP ipsilateral	Kolmogorow-Smirnow	Kruskal-Wallis	Mann-Whitney U test	p<0.05
Figure 1 b	PLP contralateral	Kolmogorow-Smirnow	Kruskal-Wallis	Mann-Whitney U test	p<0.05
Figure 1 c	Iba1 ipsilateral	Kolmogorow-Smirnow	Kruskal-Wallis	Mann-Whitney U test	p<0.05

Figure 1 d	Iba1 contralateral	Kolmogorow-Smirnow	Kruskal-Wallis	Mann-Whitney U test	$p<0.05$
Figure 1 e	Caspase3 ipsilateral	Kolmogorow-Smirnow	Kruskal-Wallis	Mann-Whitney U test	$p<0.05$
Figure 1 f	NeuN ipsilateral	Kolmogorow-Smirnow	Kruskal-Wallis	Mann-Whitney U test	$p<0.05$
Figure 1 g	Serum NfL	Kolmogorow-Smirnow	Kruskal-Wallis	Mann-Whitney U test	$p<0.05$
Figure 3 a	Cu++oxLDL	Kolmogorow-Smirnow	Kruskal-Wallis	Mann-Whitney U test	$p<0.05$
Figure 3 b	HOCloxLDL	Kolmogorow-Smirnow	Kruskal-Wallis	Mann-Whitney U test	$p<0.05$
Figure 3 c	PPm	Kolmogorow-Smirnow	Kruskal-Wallis	Mann-Whitney U test	$p<0.05$
Figure 3 d	TAC	Kolmogorow-Smirnow	Kruskal-Wallis	Mann-Whitney U test	$p<0.05$
